# *Plasmodium falciparum* genetic diversity; implications for malaria control in Ethiopia: Systematic review and meta‐analysis

**DOI:** 10.1002/hsr2.70092

**Published:** 2024-09-29

**Authors:** Zufan Y. Abriham, Aysheshim K. Belew, Lemlem D. Baffa, Berhanu Mengistu, Moges Gasahw, Esmeal A. Mohammod, Muluken C. Agimas, Mekonnen Sisay, Dessie A. Angaw

**Affiliations:** ^1^ Department of Medical Parasitology, School of Biomedical and Laboratory Sciences College of Medicine and Health Sciences, University of Gondar Gondar Ethiopia; ^2^ Department of Human Nutrition, Institute of Public Health College of Medicine and Health Sciences, University of Gondar Gondar Ethiopia; ^3^ Department of Physiotherapy, School of Medicine College of Medicine and Health Sciences, University of Gondar Gondar Ethiopia; ^4^ Department of Epidemiology and Biostatistics, Institute of Public Health College of Medicine and Health Sciences, University of Gondar Gondar Ethiopia

**Keywords:** genetic diversity, meta‐analysis, multiplicity of infection, P. falciparum, systematic review

## Abstract

**Background:**

In malaria endemic regions, *Plasmodium falciparum* infection is characterized by variable genetic diversity at different settings. The parasite's various forms are found at varied frequency in different geographic areas. Understanding malaria parasite diversity and transmission is vital to evaluate control interventions. The aim of this study was under taken to determine the status of *P. falciparum* genetic diversity and MOI in different regions of Ethiopia.

**Methods:**

Relevant publications were identified from electronic databases such as; PubMed, EMBASE, Google scholar and Google. Besides, an online search was done using the above databases for all articles published in English on genetic diversity of *P. falciparum* in Ethiopia. STATA software was used for data analysis. The pooled estimates were calculated using random effect model. The summary estimates were presented using forest plots and tables.

**Results:**

A total of 11 studies were included in the systematic review. However, only 8, 10 and 2 studies were included for Pfmsp‐1, Pfmsp‐2 and glurp gene meta‐analysis, respectively. However, the meta‐analysis result showed that the pooled prevalence of Pfmsp‐1, msp‐2 and glurp gene were 84% for both msp‐1/2% and 51%, respectively. The pooled prevalence of msp‐1 gene was higher in Amhara followed by Oromia region and lower in SNNPR while, for msp‐2 gene the pooled prevalence was higher in Benshangul gumez region. Among the allelic family of msp‐1 and msp‐2 genes, MAD20 (34%) and FC27 (44%) were the most predominant respectively.

**Conclusion:**

Based on the review, there is evidence of the presence of high genetic diversity of *P. falciparum* parasites in Ethiopia, suggesting that malaria transmission remain high and that strengthened control efforts are needed. The approaches and methods used for investigation of diversified parasites have similarity between studies and should use advanced molecular techniques, like microsatellite, to assess the genetic diversity of *P. falciparum* for better results.

## 1 INTRODUCTION

Malaria is a disease caused by protozoan parasites of the genus *Plasmodium* and transmitted by female Anopheles mosquitoes.^1^ Malaria remains an important global public health problem.^2^ According to the World Health Organization^3^ report, there were an estimated 247 million malaria cases in 2021 in 84 malaria endemic countries, an increase from 245 million in 2020 at 85 malaria endemic countries in the world.^3^, ^4^ Of which the WHO Africa region accounts for about 95% of cases and 96% of deaths globally; 80% of all deaths in this region are among children aged under 5 years.^4^ *Plasmodium falciparum* is responsible for the majority of malaria infections.^5^ In Ethiopia *P. falciparum* and *Plasmodium vivax* are the main species accounting for roughly 65% and 34% of malaria cases respectively.^6^

*P. falciparum* has a considerable genetic variation in different malaria parasite isolate groups.^7^ As a result, *P. falciparum* produces life‐threatening disease and poses a global challenge to the development of effective medications, diagnostic tools, and vaccinations.^8^ People residing in high malaria transmission intensity areas can be infected with many and genetically diverse clones of *P. falciparum* at the same time, resulting in multiplicity of infection (MOI).^9^ Genetically varied and multiple *P. falciparum* infections occur in both asymptomatic and symptomatic malaria patients.^10^, ^11^ *P. falciparum* genetic diversity and MOI increase parasite virulence, malaria pathogenesis and immune evasion.^12^

*P. falciparum* has a long evolutionary history with its human host and has a lot of genetic variety, particularly in the surface antigens which have been subjected to a lot of selective pressure from the human immune system and have been the focus of subunit vaccines.^13^ In response to medications and other malaria interventions, the parasite continues to change through mutation and sexual recombination, making these interventions need to be changed.^14^ When malaria vaccinations are employed, malaria parasite that circumvent vaccine are likely to evolve, posing a danger to vaccine efficacy.^14^

Due to antigenic variation in *P. falciparum*, numerous alleles significantly decrease vaccine efficacy, effectively avoiding vaccine‐induced allele specific immunity.^14^ Therefore, information on genetic diversity of *Plasmodium* parasites is critical in producing an effective malaria vaccine.^14^ In endemic situations, high recombination rates among different *P. falciparum* clones result in the creation of much diversified parasite isolates as well as the rapid emergence and spread of drug‐resistant *P. falciparum* parasite strains.^15^ The rise of antimalarial drug resistance in control programs, maintaining a diversified parasite population in the face of decreasing prevalence would remain an important public health concern.^14^

There are several polymorphic gene markers in *P. falciparum* isolates for genotyping, such as the merozoite surface protein‐1 and −2 (msp‐1 and msp‐2) genes and the glutamate‐rich protein (glurp) gene.^16^ msp‐1 and msp‐2 are *P. falciparum* blood‐stage malaria vaccine targets.^17^ msp‐1 is major surface protein encoded by msp‐1 on chromosome 9, which contains 17 blocks of sequences flanked by conserved regions.^18^ It plays a major role in erythrocyte invasion and is targeted by immune responses.^19^ Block 2, which is the most polymorphic part of msp‐1, is grouped under three allelic families of K1, MAD20 and RO33.^20^

msp‐2 is an abundant protein on the surface of *P. falciparum* merozoite.^21^ The gene is located on chromosome 2 and is composed of five blocks the most polymorphic of which is the central block 3 encoded by highly divergent alleles grouped into two dimorphic families, FC27 and 3D7. msp‐2 is characterized by highly polymorphic central repeats domains.^22^ The glurp being a potential vaccine candidate has been tested in Phase I trial of vaccine development^23^ and is expressed in both the erythrocyte stages of the parasite life cycle.^24^ Glurp protein‐based antibodies can inhibit the growth of *P. falciparum* and are suggested to play important role in controlling parasitaemia.^25^

The characterization of *P. falciparum* genetic diversity and MOI aids in determining if leftover residual historical parasite lineages contribute to local malaria transmission, or if new parasite lineages migrating from other locations contribute to contemporary malaria transmission.^26^ It also helps to determine gene flow dynamics and provides insight into the appropriate malaria control interventions.^27^ Thus, this review aims to assess the status of *P. falciparum* genetic diversity and MOI in different regions of Ethiopia.

## 2 METHODS

### 2.1 Search strategy

Systematic review and meta‐analysis were conducted based on preferred reporting items for systematic reviews and meta‐analysis guidelines (PRISMA).^28^ The PRISMA checklist was perform to ensure that all relevant information that were included in the analysis. The protocol for this review has been submitted to PROSPERO and assigned the registration number CRD42023408293. To find potential studies for this systematic review and meta‐analysis by using different databases to retrieve data, the literature search was conducted by two reviewers using Medical Subject Heading (MeSH) terms/key words. A pilot search in the related MeSH terms was conjoined with the Boolean operator “OR” and the unrelated MeSH terms were conjoined with the Boolean operator “AND” to form a search string. The search string was applied in all databases namely PubMed, EMBASE, Google scholar, and advanced Google to search for the articles. An example of keywords used in databases to select relevant studies will as follows: (“genetic diversity”) OR “genetic variation” OR “genetic polymorphism” OR Genotype AND (“*P. falciparum*”) OR “*P. falciparum*” AND “multiplicity of infection” OR “complexity of infection” AND Ethiopia and all searches will restrict to paper titles/abstracts (Table S1). The Endnote software version X9 was used to manage references and remove duplicated references.

### 2.2 Eligibility criteria

#### 2.2.1 Inclusion criteria and exclusion criteria

All studies that report on *P. falciparum* genetic diversity and MOI among individuals of all age groups and gender in Ethiopia were included. Observational studies (cohort, cross‐sectional studies) and studies published from 2015 to 2022 were included in this review. Studies that used the appropriate molecular genotyping procedures (DNA extraction, DNA amplification and fragment analysis) in determining *P. falciparum* genetic diversity and MOI were also included. Studies reporting on other *Plasmodium* species and articles whose full text cannot be retrieved were excluded.

#### 2.2.2 Screening of articles

The searched articles were assessed for the outcomes of interest in any part of Ethiopia. The screening process was guided by the updated PRISMA screening guidelines.^28^ Although this systematic review and meta‐analysis was reported based on the above guideline. (Table S2) The articles were undergoing title and abstract screening and those that pass these criteria was considered for full‐text screening. The articles that have undergone full‐text screening were the ones to include in this review. The screening was done in duplicates by two independent reviewers. Disagreements were resolved through discussion between a pair of other independent reviewers to reach a consensus.

#### 2.2.3 Data extraction

Data extraction were done on articles that have passed full‐text screening criteria by two reviewers using excel spreadsheet form and evaluated by the third reviewer. From each study, the following parameters were extract, name of the first author, year of publication, study area (region), study design, sample size, study review outcomes namely, *P. falciparum* msp‐1, msp‐2, glurp gene with their allelic family, MOI, type of the diagnostic method used and genotyping approaches. The extracted data was independently cross‐checked by two reviewers.

### 2.3 Study selection and quality assessment

Studies were assessed for quality, with only high‐quality studies included in the analysis. The quality of included studies was also assessed using adapted version of Newcastle‐Ottawa Scale (NOS). Two authors independently assess the methodological quality, quality of reported data (extractable data to calculate the pooled prevalence of msp‐1, msp‐2 and glurp gene of *P. falciparum* with their family). The criteria include 3 categories with maximum score of 9 points; the first is “selection” category which accounts a maximum of 4 points, the second is “comparability” category which accounts a maximum of 2 points, and the third is “outcome” which accounts a maximum of 3 points. Based on these classifications, studies with a composite score ≥7 were considered as “good” quality study and all included studies in this review were considered as “good” quality study (File S2).

### 2.4 Data analysis

The data synthesis was done independently for each primary outcome. Heterogeneity was assessed by using Higgins et al. method and quantified by calculating the I.^2^ The values of I^2^ 25%, 50%, and 75% were assumed to represent low, medium, and high heterogeneity, respectively. Heterogeneity was observed and a separate forest plot was constructed based on the source of heterogeneity. Funnel plot and Eggers's test were used to assess for publication bias. *p* < 0.05 was considered indicative of statistically significant publication bias. To minimize the variance of point estimates between primary studies sub‐group analysis was conducted based on the type of marker used, regions, and year of publication. The data was analyzed using STATA 17 software.

## 3 RESULTS

### 3.1 Study selection and identification

**Figure 1:**
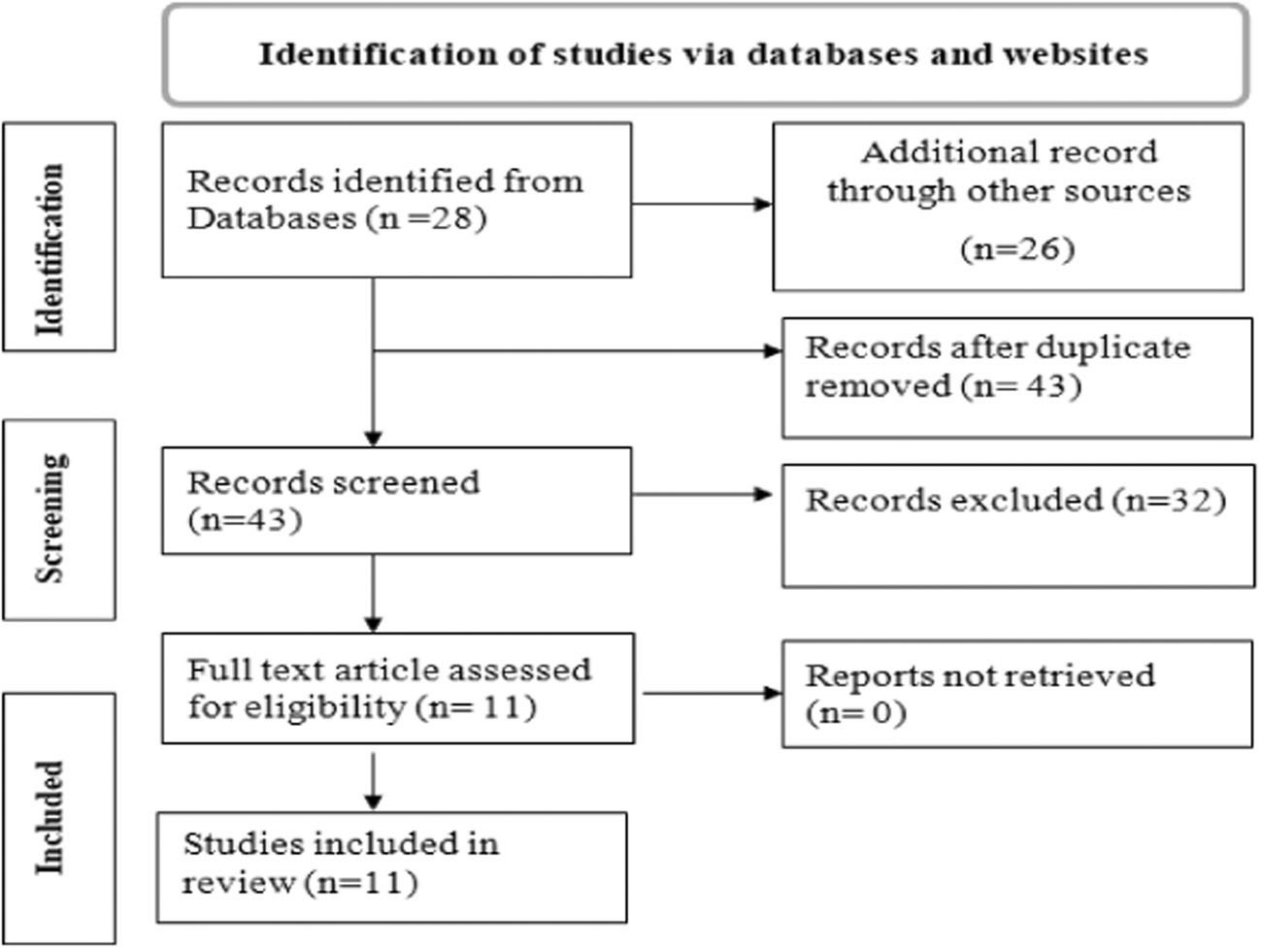
PRISMA flow diagram of article selection for systematic review and meta‐analysis of *Plasmodium falciparum* genetic diversity. PRISMA, preferred reporting items for systematic reviews and meta‐analysis.

This review includes published articles on *P. falciparum* genetic diversity and multiplicity of infection in Ethiopia. We used PubMed, EMBASE, Google Scholar, and Google searching databases to find potential articles for this systematic review and meta‐analysis. A total of 54 articles were identified through database search. After removing duplicates 43 articles were left. By further screening on reading title and abstract, 32 duplicate articles were excluded; finally, 11 potential articles were included for qualitative and quantitative synthesis for this review (Figure 1).

### 3.2 Characteristics of included studies

A total of 11 studies were included for this systematic review and meta‐analysis. Among the included studies 8 (72.70%) were published after 2018. All the included articles were cross‐sectional surveys, of which one studies community based and 10 studies health facility‐based based cross‐sectional surveys. The total sample size for studies was 1165 study participants age range of 6 month—80 years. The minimum and maximum sample size was reported 50 and 225 respectively. Out of the 11 studies, five studies were from Oromia region (Beset, surrounding Adama, Chewaka district, Anger Gute and warabo, and Metehara) and three studies were from Benshangul gumez region. The remaining three studies were from Amhara region, SSNPR and Afar region respectively. The dry blood spot samples in the studies were collected from uncomplicated and sever *P. falciparum* patients and the diagnosis methods for *P. falciparum* were microscopy in nine studies and both microscopy and qPCR in one study while, only qPCR in the remaining one study. Chelex‐100® DNA extraction method was used in most of the studies and left two studies was used Proteinase k and Qiagen extraction techniques.

All the included studies for assessing the status of *P. falciparum* genetic diversity and MOI were determined through genotyping of the polymorphic regions of the block 2 of merozoite surface protein‐1 (msp‐1), block 3 of merozoite surface protein‐2 (msp‐2) and the RII repeated region of the glutamic rich protein (glurp). Among these studies both msp‐1 and msp‐2 detected from five studies, msp‐1, msp‐2 and glurp detected from two studies, only msp‐1 determined from one studies and only msp‐2 marker detected from three studies. All included studies were used size polymorphism followed by gel electrophoresis for genotyping of *P. falciparum* genetic polymorphism (Table 1).

**Table 1:** Characteristics of studies included in the systematic review and meta‐analysis.

Authors	Year of publication	Region	Study population	Study design	Sample size	Sample type	Detected markers
Chekol et al.^29^	2022	Oromia & SNNPR	Uncomplicated	Cross sectional	50	DBS	msp‐1 & msp −2
File et al.^30^	2022	Oromia	Uncomplicated	Cross sectional	148	DBS	msp −2
Mohammed et al.^2^	2017	Benshangul gumez	Uncomplicated	Cross sectional	92	DBS	msp −2
Mohammed et al.^31^	2015	SNNPR	Uncomplicated	Cross sectional	88	DBS	msp −1 & msp −2
Mohammed et al.^32^	2018	Amhara	Uncomplicated	Cross sectional	90	DBS	msp −1, msp −2& glurp
Mohammed et al.^18^	2019	Benshangul gumez	Uncomplicated & sever	Cross sectional	118	DBS	msp −1 & msp −2
Abamecha et al.^10^	2020	Oromia	Uncomplicated	Cross sectional	80	DBS	msp −1 & msp −2
Mohammed et al.^33^	2021	Afar	Uncomplicated	Cross sectional	52	DBS	msp −2
File et al.^30^	2021	Oromia	Uncomplicated	Cross sectional	139	DBS	msp −1
Tadele et al.^34^	2022	Benshangul gumez and Oromia	Uncomplicated	Cross sectional	225	DBS	msp −1 & msp −2
Reda et al.^35^	2022	Oromia	Uncomplicated	Cross sectional	70	DBS	msp‐1, msp‐2 & glurp

Among the included studies the relationship between age and parasitemia with MOI was done. However, most of the studies indicate there is no significant relation between MOI with both age and parasite density of the participant and some of the remaining studies detect direct and indirect relation of age and parasitemia of participants with MOI. The number of different *P. falciparum* strains co‐infecting a single host or MOI was calculated from each studies and the overall MOI was range from (1.1−3.2).

### 3.3 Quality assessment

All studies were evaluated with criteria of adapted version of Newcastle‐Ottawa Scale (NOS) tool for these studies included in this review. The result of the analysis showed that the included studies had low risk of bias because its total score is >7 which is considered as good quality of studies.

#### 3.3.1 Pooled prevalence of msp‐1, msp‐2 and glurp gene of *P. falciparum*

**Figure 2:**
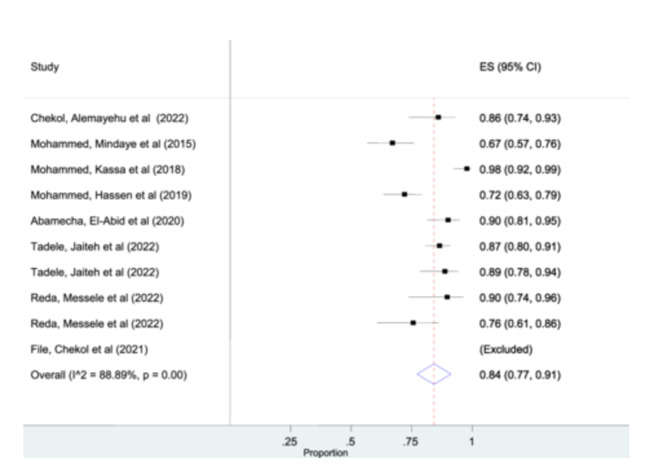
Forest plot representing pooled estimates msp‐1gene across studies from different parts of Ethiopia.

Out of 11 published articles that are included in this systematic review, eight studies were included for the overall estimation of msp‐1 gene of *P. falciparum*. One study was excluded by the STATA software analysis due to its outlier characteristics. The estimated pooled prevalence of msp‐1 gene using random effects model was found to be 84% (95% CI: 77%– 91%) (Figure 2).

**Figure 3:**
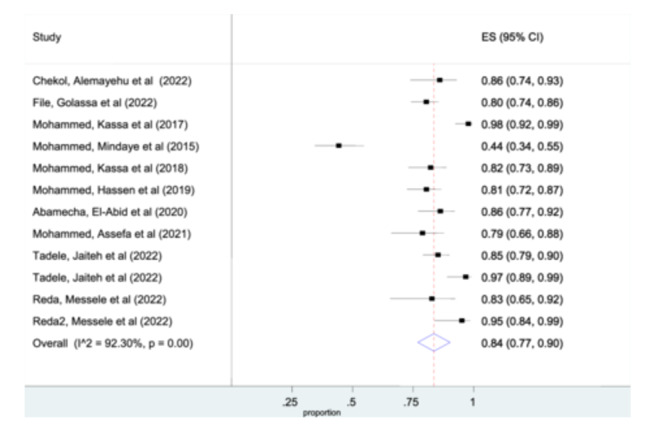
Forest plot representing pooled estimates msp‐2 gene, across studies from different parts of Ethiopia.

Among the included studies, ten studies were included for the pooled prevalence of msp‐2 gene analysis. The pooled prevalence of individuals in msp‐2 gene was 84% (95% CI: 77%–90%) (Figure 3). However, glurp detected from only two studies and the pooled prevalence of glurp analysis was 51% (95% CI: 41%–60%). (Figure S1).

#### 3.3.2 Pooled prevalence of allelic family of msp‐1 and msp‐2 gene of *P. falciparum* and it polyclonal infection

The prevalence of allelic family of msp‐1 (K1, MAD20, and RO33) and msp‐2 (FC27 and 3D7) gene were detected studies in Ethiopia. All of the studies included in this review reported the prevalence of each allelic family. msp‐1 has three different allelic family such as (K1, MAD20, and RO33) and the pooled prevalence of each allelic family were 23% (95% CI: 14%–32%), 34% (95% CI: 17%–52%), and 14% (95% CI: 9%–20%) respectively. On other hand, msp‐2 has two allelic family, (FC27 and 3D7), their pooled prevalence was 44% (95% CI: 20%–67%) and 26% (95% CI: 17%–35%) respectively. While the prevalence of mixed (polyclonal) infections was reported in all studies, overall, the pooled prevalence of msp‐1 allelic family was as follows: K1 + MAD20 15% (95% CI: 10%–20%), K1 + RO33 10% (95% CI: 5%–15%), RO33 + MAD20 7% (95% CI: 3%–10%) and K1 + RO33 + MAD20 9% (95% CI: 4%–14%). The pooled prevalence of mixed infection for msp‐2 (FC27 + 3D7) allelic family was 43% (95% CI: 27%–59%).

#### 3.3.3 Subgroup analysis of *P. falciparum* genetic diversity by region, marker type and year of publication

**Figure 4:**
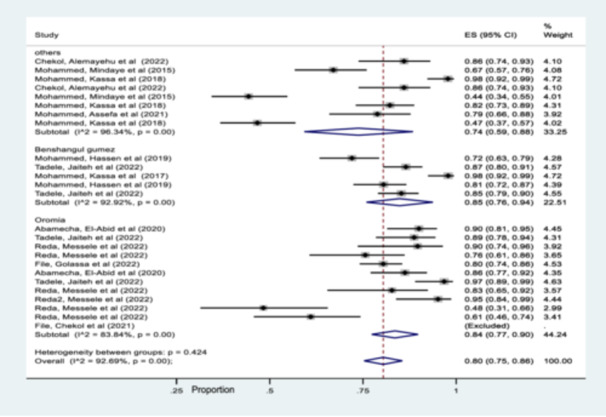
Forest plot representing subgroup analysis of pooled msp‐1, msp‐2 & glurp gene by region.

Subgroup analysis based on region showed that the pooled prevalence of msp‐1, msp‐2 and glurp gene in Oromia, Benshangul gumez, and others including (SNNPR, Amhara, and Afar) were 84% (95% CI 77%–90%), 85% (95% CI 76%–94%), and 74% (95% CI 59%–88%), respectively (Figure 4).

**Figure 5:**
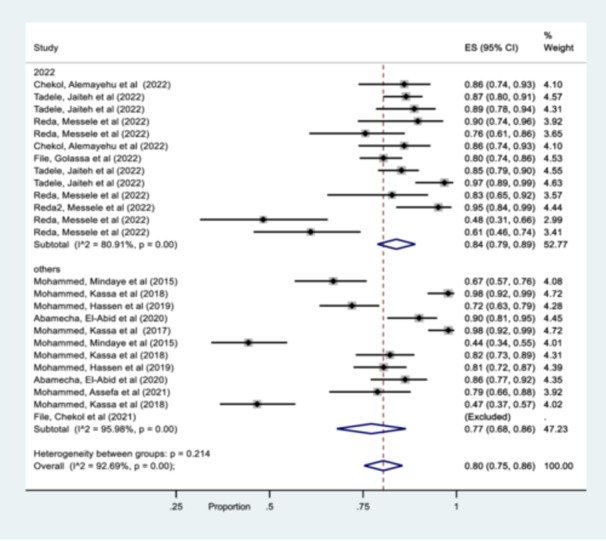
Forest plot representing subgroup analysis of pooled msp‐1, msp‐2 & glurp gene by year of publication.

Similar pattern was also observed year of publication analysis and therefore 84% of the studies was published in 2022, while 77% was punished in other years (2015–2021) (Figure 5).

This meta‐analysis study also showed that the pooled estimated values based on marker type, of them both msp‐1 & msp‐2 gene detected in the included studies was 81% and for msp‐1, msp‐2 & glurp genes the pooled estimated value was 76%. However, the pooled estimated values of only msp‐2 marker were 86% while, studies that detected only msp‐1 marker was excluded due to its outlier characteristics. The summary estimates based on the different types of genotype marker is presented in Table 2.

**Table 2:** Summary estimates for pooled genotype marker based on marker types.

Type of genotype marker	Number of studies included	Pooled estimate (95% Cl)	I ^2^ (%)
MSP‐2	3	86 (72, 100)	‐
MSP‐1& MSP‐2	5	81 (75, 88)	90.38
MSP‐1, MSP‐2 & glurp	2	76 (62, 88)	94.26

### 3.4 Heterogeneity and publication bias

The existence of heterogeneity and publication bias was determined within the included studies. Consequently, there were considerable heterogeneity across the included studies in this meta‐analysis (I^2^ = 92.69%, *p*‐value < 0.001). The highest weight among studies was observed from studies conducted by Mohammed, et al.,^32^ Mohammed, et al.,^2^ Tadele, et al.^34^ The included studies were assessed for potential publication bias using Egger's test.

### 3.5 Sensitivity analysis

From the random effect model, there are no studies excessively influenced the overall prevalence of msp‐1, msp‐2 and glurp pooled estimate among the included studies. (Figure S2).

## 4 DISCUSSION

Most malaria cases identified in this country are caused by *P. falciparum.* The extensive genetic polymorphism of this parasite gives it the capacity to evolve multidrug resistance or evade vaccination. Malaria control efforts are hampered by the introduction and spread of resistant strains and the situation is getting worse in some areas.^36^, ^37^ A better understanding of the population structure of *P. falciparum* genotypes can be an important element in adjusting control strategies for this parasite in the country. Beyond measuring transmission intensity, understanding the extent of polyclonal infections is critical for molecular malaria surveillance activities. Drug resistance molecular marker typing is an important part of malaria monitoring.^38^ In Ethiopia, different studies were used PCR genotyping analysis with the polymorphic markers msp‐1, msp‐2 and glurp were performed to gain insight into the genetic diversity of the populations of *P. falciparum* parasite species in different parts of Ethiopia.

Evidence on the pooled estimates of msp‐1, msp‐2 and glurp gene is limited in the Ethiopia context. Based on the individual articles that are included under this review, the level of msp‐1 gene across malaria endemic setting ranged from the highest 98% (in Amhara region) to the lowest 67% (locality in SNNPR).^31^, ^32^ However, this one outlier individual studies were not included in the meta‐analysis. This study showed that, the pooled prevalence of msp‐1 gene was 100% (surrounding Adama).^29^ Moreover, the overall prevalence of *P. falciparum* with msp‐1 gene is high (84%), even though some of the individual studies have not detected the msp‐1 gene.

On the other hand the level of msp‐2 gene across different setting ranged from the lowest 44% (in SNNPR) to the highest 98% (in Benshangul gumez).^2^, ^31^ The overall prevalence of *P. falciparum* with msp‐2 gene is similar and high (84%) as msp‐1 gene, while some of the individual studies did not confirm the msp‐2 gene (Table 1). Supporting the conclusions of the majority of studies undertaken studies in Kinshasa Province, Democratic Republic of Congo,^39^ This confirms that in many malaria‐endemic African nations, polymorphism pfmsp2 allelic families may be circulating more often.

Even though, the density of the malaria parasite in human blood is believed to have a strong correlation with the GLURP gene detectability, and sickness severity, the genetic diversity of GLURP has also investigated in Ethiopia, from this review, two studies were detected for this gene and with the pooled prevalence of 51%.^32^, ^35^ Similarly, there are reports from other African countries which detects the glurp gene such as in Kinshasa, DRC.^40^

This systematic review and meta‐analysis study showed that the pooled prevalence of each allelic family of msp‐1 (K1, MAD20, and RO33) and msp‐2 (FC27 and 3D7) were detected studies in Ethiopia. Among msp‐1 allelic family such as (K1, MAD20, and RO33) the pooled prevalence of MAD20 (34%) was higher than other allelic family K1 (23%) and RO33 (14%) respectively. This indicates that MAD20 is the most predominant allelic families from msp‐1 gene at different part of Ethiopia. This difference may be due to mutation, genetic recombination, and geographical variation sample size of study population.

On other hand, from msp‐2 gene which has two allelic family, (FC27 and 3D7), the pooled prevalence was higher in FC27 (44%) than 3D7 (26%). This shows that the distribution of FC27 allele is highly dominant than 3D7 allele in different regions of Ethiopia. This could be due to the variation in geographical locations, use of different antimalaria drugs and size of the study sample population.

Polyclonal infections are the most frequently reported measures of malaria genomic epidemiology studies and have been proposed as surrogate markers for transmission intensity this result from the bite of mosquitoes infected with more than one clone or from multiple bites.^41^ The prevalence of mixed (polyclonal) infections was reported in all studies, overall the pooled prevalence of msp‐1 allelic family was as follows: K1 + MAD20 (15%), K1 + RO33 (10%), RO33 + MAD20 (7%) and K1 + RO33 + MAD20 (9%). The pooled prevalence of mixed infection for msp‐2(FC27 + 3D7) allelic family was 43%.

In this study, there was a significant heterogeneity among studies and hence a subgroup analysis was carried out. Accordingly, the region‐wise subgroup analysis showed that the pooled prevalence of msp‐1, msp‐2 and glurp gene are higher in Benshangul gumez and Oromia than other regions (SNNPR, Amhara, and Afar). The observed differences might be associated with the study period, the number of studies done and the level of parasite transmission intensity. Similar subgroup pattern was also observed year of publication analysis and therefore 84% was published in 2022, while 77% was punished in other years (2015–2021). This meta‐analysis study also showed that the pooled estimated values of both msp‐1& msp‐2 gene detected in the included studies was 81% and for msp‐1, msp‐2 & glurp genes the pooled estimated value was 76%. However, the pooled estimated values of only msp‐2 marker was 86% while, studies that detected only msp‐1 marker was excluded due to its outlier characteristics.

Age is considered as important factor in the acquisition of immunity against *P. falciparum* and may have also an effect on MOI, although, the influence of age on the MOI is highly affected by malaria transmission intensity.^42^, ^43^ High malaria transmission and also parasite density may have a strong association with the genetic diversity of *P. falciparum* in Ethiopia and high parasite density increase the probability of detecting concurrent clones in an individual.^44^ In this systematic review and Meta analysis all studies included in the review shows that the relationship between MOI with age and parasitemia of the participant and all the included studies expressed that there is no association between age and parasite density with MOI, and other studies shows there is negative association between MOI and age as well as parasitemia of participants. The number of different *P. falciparum* strains co‐infecting a single host, (MOI), has been shown to be a common feature in most malaria endemic areas and was reported to vary with age, parasite density, immune status, epidemiological settings and transmission intensity. Transmission intensity can affect the genetic diversity of the parasite population and high parasite density increase the probability of detecting concurrent clones in an individual.^36^, ^44^

In malaria endemic regions, *P. falciparum* infection is characterized by variable genetic diversity at different settings in Ethiopia by using different markers like MSP‐1, MSP‐2 and GLURP gene. For future studies, it would be advisable to employ more sensitive and precise genotyping methods such as capillary electrophoresis, next‐generation sequencing and microsatellite loci analyisis which were done in other African countries including Nigeria.^45^ These techniques offer higher resolution and sensitivity.

This review summarized the status of *P. falciparum* genetic diversity and multiplicity of infection among individuals in different settings in Ethiopia Understanding malaria parasite diversity and transmission dynamics is vital to as input for policymakers for developing antimalarial drug, effective eradication strategies and vaccine.^46^ Furthermore, this study is the first review on of *p. falciparum* genetic diversity and multiplicity of infection in Ethiopia. Despite this, the present study has few limitations. First, a small number of articles included in this systematic review and meta‐analysis could affect the pooled prevalence estimate. Second, more than half of the included articles were obtained from the Oromia Regional State, whereas the other was found to be in Amhara, SNNPR, Benshangul gumez and Afar regional states and unequal distribution of articles throughout the country may affect the outcomes of this study. Third, no study obtained from Gambella, Harari, and Tigray regions. Therefore, mentioned limitations might affect the results reported in this review regarding the overall prevalence *P. falciparum* genetic diversity and multiplicity of infection in Ethiopia. In addition, all the included articles were used PCR products analysis followed by gel electrophoresis. Due to the limits of the technique employed, it's possible that the total numbers of alleles were underestimated and may hamper comparison of study outcomes.

This review implies, the study showed high levels of genetic diversity and mixed strain infections of *P. falciparum* populations in different part of Ethiopia, suggesting that malaria transmission remain high and that strengthened control efforts are needed. Furthermore, the identification of malaria hotspot areas is important for the implementation of targeted interventions. In addition, study findings will serve as baseline data for future studies on parasite population structure and antimalarial drug resistance surveillance across Ethiopia.

## 5 CONCLUSION

This systematic review and meta‐analysis showed that there is a high prevalence of msp‐1 and msp‐2 gene indicating the impending challenges in the use of this crucial tool in malaria control program. High genetic diversity was found on parasite strains reflecting the decline of malaria transmission interventions effectiveness. This review shows the high level of polyclonal infections with *P. falciparum* parasites harboring multiple genotypes and also infections with high MOI indicate the extensive genetic diversity and complexity of *P. falciparum* infection in different endemicity settings across Ethiopia.

## AUTHOR CONTRIBUTIONS

Zufan Y. Abriham develop the concept of the review and involved in screening, searching of the literature, data extraction, analyzed the results and writing the manuscript. Aysheshim K. Belew, Lemlem D. Baffa, Berhanu Mengistu, Moges Gasahw, Esmeal A. Mohammod, Muluken C. Agimas, Mekonnen Sisay and Dessie A. Angaw were involved in the searching, screening of the literature, data extraction and writing the manuscript. Also all authors have read and approved the final version of the manuscript and Zufan Y. Abriham had full access to all of the data in this study and takes complete responsibility for the integrity of the data and the accuracy of the data analysis.

## CONFLICT OF INTEREST STATEMENT

The authors declare no conflict of interest.

## ETHICS STATEMENT

All participants provided written informed consent to publish their data in the individual studies.

## PROTOCOL REGISTRATION

This review protocol has been submitted to PROSPERO and registered with the number CRD42023408293.

## TRANSPARENCY STATEMENT

Zufan Yiheyis Abriham affirms that this manuscript is an honest, accurate, and transparent account of the study being reported; that no important aspects of the study have been omitted; and that any discrepancies from the study as planned (and, if relevant, registered) have been explained.

## Supporting information

Supporting information.

Supporting information.

Supporting information.

Supporting information.

Supporting information.

## Data Availability

We confirm that all the data for this manuscript are available and can be shared upon request and if someone wants to request the data from this study it is directed to the first author (Zufan Y. Abriham).
