# Supplementary material for: Plasmodium falciparum genetic diversity; implications for malaria control in Ethiopia: Systematic review and meta‐analysis
Source: Health Sci Rep. 2024 Sep 29;7(10):e70092. doi: 10.1002/hsr2.70092 (PMC11439746; doi:10.1002/hsr2.70092)
Supplement: Supplementary file 1 — Supporting information. [file HSR2-7-e70092-s002.docx]

| Search | Search terms | Hits |
| --- | --- | --- |
| 1 | **(((genetic diversity[Text Word]) OR (genetic variation[Text Word])) OR (genetic polymorphism[Text Word])) OR (genotype[Text Word])** | 505,996 |
| 2 | **(((plasmodium falciparum[Text Word]) OR (p. falciparum[Text Word])) OR (Plasmodium falciparum[Text Word])) OR (P.falciparum[Text Word])** | 46,257 |
| 3 | #1 OR #2 | 2,655 |
| 4 | **(((multiplicity of infection[Text Word]) OR (complexity of infection[Text Word])) OR (MOI[Text Word])) OR (COI[Text Word])** | 16,009 |
| 5 | #3 AND #4 | 210 |
| 6 | **Ethiopia[Text Word]** | 31,796 |
| 7 | **((((((genetic diversity[Text Word]) OR (genetic variation[Text Word])) OR (genetic polymorphism[Text Word])) OR (genotype[Text Word])) AND ((((plasmodium falciparum[Text Word]) OR (p. falciparum[Text Word])) OR (Plasmodium falciparum[Text Word])) OR (P.falciparum[Text Word]))) AND ((((multiplicity of infection[Text Word]) OR (complexity of infection[Text Word])) OR (MOI[Text Word])) OR (COI[Text Word]))) AND (Ethiopia[Text Word])** | 14 |

Table S1: PubMed search strategy for genetic diversity of P. falciparum
