# Supplementary material for: Plasmodium falciparum genetic diversity; implications for malaria control in Ethiopia: Systematic review and meta‐analysis
Source: Health Sci Rep. 2024 Sep 29;7(10):e70092. doi: 10.1002/hsr2.70092 (PMC11439746; doi:10.1002/hsr2.70092)
Supplement: Supplementary file 2 — Supporting information. [file HSR2-7-e70092-s001.docx]

Overall (I^2 = 19.21%, p = 0.29)

Reda, Messele et al (2022)

Reda, Messele et al (2022)

Study

Mohammed, Kassa et al (2018)

0.51 (0.42, 0.60)

0.48 (0.31, 0.66)

0.61 (0.46, 0.74)

ES (95% CI)

0.47 (0.37, 0.57)

0.51 (0.42, 0.60)

0.48 (0.31, 0.66)

0.61 (0.46, 0.74)

ES (95% CI)

0.47 (0.37, 0.57)

.25

.5

.75

1

proportion

Figure S1: Forest plot representing pooled estimates of glurp across studies from different parts of Ethiopia
