# Supplementary material for: Plasmodium falciparum genetic diversity; implications for malaria control in Ethiopia: Systematic review and meta‐analysis
Source: Health Sci Rep. 2024 Sep 29;7(10):e70092. doi: 10.1002/hsr2.70092 (PMC11439746; doi:10.1002/hsr2.70092)
Supplement: Supplementary file 3 — Supporting information. [file HSR2-7-e70092-s003.docx]

Figure S2: Sensitivity analysis for prevalence of msp-1/2 and glurp gene estimation
